# An Equine Wound Model to Study Effects of Bacterial Aggregates on Wound Healing

**DOI:** 10.1089/wound.2018.0901

**Published:** 2019-08-21

**Authors:** Elin Jørgensen, Lene Bay, Lene T. Skovgaard, Thomas Bjarnsholt, Stine Jacobsen

**Affiliations:** ^1^ Department of Veterinary Clinical Sciences, Faculty of Health and Medical Sciences, University of Copenhagen, Taastrup, Denmark.; ^2^ Department of Immunology and Microbiology, Faculty of Health and Medical Sciences, University of Copenhagen, Copenhagen N, Denmark.; ^3^ Section of Biostatistics, Department of Public Health, University of Copenhagen, Copenhagen K, Denmark.; ^4^ Department of Clinical Microbiology, Rigshospitalet, Copenhagen Ø, Denmark.

**Keywords:** equine, wound, biofilm, bacterial aggregates, chronic, model

## Abstract

**Objective:** Relevant animal models to study effects of bacterial aggregates on wound healing are lacking. We aimed at establishing an equine wound model with bacterial aggregates to investigate the impact of bacterial inoculation on normal (thorax) and impaired (limb) wound healing.

**Approach:** Wounds were created on three limbs and both thorax sides of six horses. Twelve out of 20 wounds per horse were inoculated with 10^4^ *Staphylococcus aureus* and 10^5^ *Pseudomonas aeruginosa* on day 4. Healing was monitored until day 27 by clinical assessment, including wound scoring, surface pH measurements, and digital photography for area determination. Biopsies were used for bacterial culture and for peptide nucleic acid fluorescence *in situ* hybridization to detect bacterial aggregates.

**Results:** Inoculated limb wounds healed slower than noninoculated limb wounds from day 10 onward (*p* < 0.0001). Inoculated and noninoculated thorax wounds healed equally well and faster than limb wounds. The odds ratio of detecting bacterial aggregates in inoculated limb wounds was 7.1 (2.4–21.0, *p* = 0.0086) compared with noninoculated limb wounds and 36.2 (3.8–348, *p* = 0.0018) compared with thorax wounds.

**Innovation:** This equine wound model with bacterial aggregates might be superior to other animal wound models, as both normal and impaired healing can be studied simultaneously. In this model, many aspects of wound healing, including novel treatments, may be studied.

**Conclusions:** The impaired healing observed in inoculated limb wounds may be related to the persistent bacterial aggregates. Both in capability of clearing inoculated bacteria from the wounds and in healing pattern, thorax wounds were superior to limb wounds.

**Figure:**
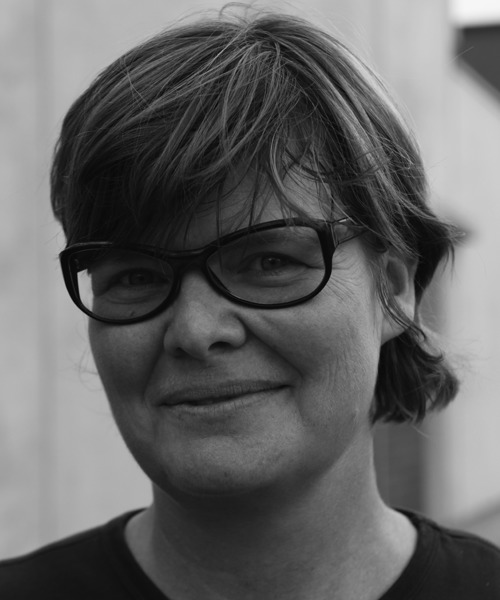
**Stine Jacobsen, DVM, PhD, Dipl ECVS**

## Introduction

Bacterial aggregates, also referred to as biofilms, are a well-known major contributor to impaired wound healing.^1–3^ Wound healing is a highly complex process that is difficult to study in controlled human studies due to the heterogeneity of wounds and patients. To gain knowledge, many animal models have been used to investigate the relationship between bacterial aggregates and wound healing.^4^ Most studies use small rodents to model wound healing. However, skin anatomy and healing patterns in these species are markedly different from those found in humans.^4^ Similar to humans, horses have tight and thick skin.^5,6^ Contrary to the situation in loose-skinned animals such as rodents, contraction of wound edges play a small role (20–25%) in extremity/limb healing in humans and horses.^7,8^ Due to the similarities in skin anatomy and relative importance of contraction and epithelialization in healing, horses seem to be valid model animals for wound healing in humans.^9,10^ Horses are prone to impaired wound healing and chronic wound formation, in particular when wounds are located on the limbs.^8,11^

Recent research detected bacterial aggregates in equine chronic wounds and suggested that aggregates might be implicated in the chronic healing pattern frequently found in equine limb wounds,^12–15^ similar to what has been shown in humans.^2,3^ The pathophysiology underlying delayed healing in horses and humans share further similarities: impaired healing of equine limb wounds has been linked to hypoxia^16^ and chronic low-grade inflammation,^17,18^ conditions which are also present in many types of chronic wounds in humans, for example, venous stasis ulcers, diabetic foot ulcers, and pressure wounds.^19,20^

This study aimed at inoculating experimental excisional wounds in horses with *Staphylococcus aureus* and *Pseudomonas aeruginosa* to establish a model for studying effects of bacterial aggregate formation in equine limb and thorax wounds. *S. aureus* and *P. aeruginosa* were chosen to mimic common wound infections in humans,^21–23^ these bacteria are incidentally also common wound pathogens in horses.^12,13,24^

## Clinical Problem Addressed

Impaired wound healing is common and causes high health expenditures and decreased quality of life of the affected patients. Bacterial aggregates cause delayed wound healing and are present in most chronic wounds.^3^ Improved animal models are needed to study bacterial aggregates in wounds to better understand pathogenesis and to test future treatment options.

## Materials and Methods

### Horses and clinical examinations

Six mature, mixed breed geldings, 3–10 years old, weighing 430–500 kg, and standing at 149–63 cm were included in this study. Horses were found to be healthy on thorough clinical, lameness, hematological, and biochemical examinations. None of the horses had scar tissue or other dermatological diseases near any of the body locations where wounds were to be created. Before the initiation of the study, horses underwent deworming (Equimax Vet, Virbac Danmark A/S, Kolding, Denmark), vaccination against tetanus (ProteqFlu-Te, Boehringer Ingelheim, Copenhagen, Denmark), teeth floating, and hoof trimming as needed. Horses were housed in box stalls and had either access to a pen or were hand-walked (2 × 20 min) daily; they were fed *ad libitum* grass hay and concentrates according to their needs. On a daily basis, the horses underwent brief clinical examinations, pain scoring according to “The Equine Pain Scale,”^25^ and lameness examinations (using the American Association of Equine Practitioners' [AAEP] scale^26^). Furthermore, blood samples were drawn on day 2, 4, 7, 14, 21, and 27 for hematological and biochemical analyses. The experimental protocols were approved by the Large Animal Teaching Hospital Ethics Committee and by the Danish Animal Experiments Inspectorate (license no. 2016-15-0201-00981), and procedures were performed according to the Danish Animal Testing Act and EU Directive 2010/63/EU for animal experiments.

### Wound creation

On day 0, horses had wounds created on the dorsolateral aspect of both metatarsi and one randomly chosen metacarpus and on both sides of the ventral thorax, just caudal to the shoulder, over the thoracic serratus ventralis muscle. Horses were sedated with intravenous detomidine hydrochloride 1 mg/100 kg (Domosedan, Orion, Nivå, Denmark), acepromazine 4 mg/100 kg (Plegicil, Dechra Veterinary Products A/S, Uldum, Denmark), atropine sulfate 0.5 mg/100 kg (Skanderborg Apotek, Skanderborg, Denmark) and buthorphanoltartrate 2 mg/100 kg (Torbugesic, Orion, Nivå, Denmark) and the sedations were maintained with xylazine hydrochloride continuous infusion (Xylavet, ScanVet Animal Health A/S, Fredensborg, Denmark) titrated to effect. The body locations to be wounded were anesthetized with regional nerve blocks using a 1:1:1 mixture of 1.9% lidocaine hydrochloride (Lignovet, ScanVet Animal Health A/S, Fredensborg, Denmark), 0.5% bupivacaine (Marcaine, AstraZeneca, Albertslund, Denmark), and isotonic saline. Before creation of wounds, hair was clipped and skin aseptically prepared using 2 × 4 min scrub with 4% chlorhexidine gluconate (Medi-Scrub, Rovers Medical Devices B. V., Oss, Netherlands) followed by multiple applications of 70% isopropyl alcohol. Four excisional wounds were created (2 × 2 cm, 2 cm apart) in a vertical column on each of the five body locations using a scalpel and a flexible sterile template made from x-ray film. Wounds were full thickness and did not include the periosteum at the metatarsi/-carpi or the subcutaneous muscle fascia at the thorax. Wounds were photographed within 5 min of creation to measure the initial areas. All wounds were left to heal by second intention and were bandaged using sterile nonadhesive gauze (Melolin, Smith & Nephew, Hørsholm, Denmark). For the limb wounds, the dressing was secured using cotton and elastic adhesive wrap (KRUUSE Vet-Flex and KRUUSE Vet-Plast; Jørgen Kruuse A/S, Langeskov, Denmark), while on the thorax, the dressing was held in place by absorbent dressing pads (Zetuvit; HARTMANN-ScandiCare AB, Anderstorp, Sweden) and elastic adhesive wrap (KRUUSE Vet-Flex and KRUUSE Vet-Plast, Jørgen Kruuse A/S, Langeskov, Denmark). On day 0 (before surgery) and 1, horses received flunixin meglumine 1.1 mg/kg (Flunixin; ScanVet Animal Health A/S, Fredensborg, Denmark) to minimize discomfort associated with the surgical procedure. Furthermore, for the first 14 days of the study, the horses received omeprazole (Gastrogard, Merial Norden A/S, Copenhagen, Denmark) to reduce risk of gastric ulcer development.

### Wound inoculation

All four wounds on two randomly chosen limbs and one randomly chosen thorax side were inoculated with ∼10^4^ colony-forming units (CFU) *S. aureus* (clinical isolate from an equine wound) and ∼10^5^ CFU *P. aeruginosa* (PAO1 wild type)^27^ on day 4. Bacterial strains and concentrations were directed from a previous pilot study as described below.

Bacterial suspensions were prepared by inoculating freeze cultures on blood agar plates (SSI Diagnostica, Hillerød, Denmark) for 18 h at 37°C. One colony of each strain was grown in Luria–Bertani (LB) broth (Panum Institute Substrate Department, University of Copenhagen, Denmark) over night at 37°C by 180 rpm. Overnight cultures were reinoculated into LB broth and grown at 32°C by 180 rpm for further 22 h.

Bacterial suspensions were washed, resuspended in isotonic saline, adjusted to OD_600nm_ (optical density at 600 nm) 0.3 (∼10^8^ CFU/mL) and OD_600nm_ 0.4 (∼5 × 10^7^ CFU/mL) for *P. aeruginosa* and *S. aureus*, respectively, and diluted to final suspensions. The inoculation suspensions were subsequently plated and the bacterial concentrations were verified.

The suspensions were kept on ice and transported directly to the equine facility to inoculate the wounds. On day 4, 0.5 mL suspension of *S. aureus* (containing ∼10^4^ CFU) and 0.5 mL suspension of *P. aeruginosa* (containing ∼10^5^ CFU) were added simultaneously on 2 × 2 cm nonadhesive gauze that were applied to the wounds to be inoculated (12 per horse). The remaining eight wounds were treated with 1 mL isotonic saline applied to 2 × 2 cm nonadhesive gauze. After application of bacterial suspensions or isotonic saline, wounds were bandaged as described above.

### Pilot study to establish inoculation dose and timing

To establish inoculation doses and timing of application of bacteria, a pilot study was performed before the main study described above. This included one horse (5 years old warm blood gelding, 161 cm, and 480 kg). The wounding procedure for the horse was as described above, and inoculation was performed earlier, 2 days after surgery. Three different *S. aureus* strains (two clinical wound isolates and one laboratory strain) and one *P. aeruginosa* (PAO1 wild-type) were prepared as described above and applied in dosages of ∼10^6^ CFU and ∼10^7^ CFU per wound either alone or together. Eighteen out of 20 wounds were inoculated. The day after inoculation, the horse developed fever (40.1°C, normal below 38.5°C), and 2 days after inoculation, the horse showed sign of septicemia with fever, elevated heart rate, and severely elevated levels of inflammatory markers in blood. Eight days after, inoculation levels of inflammatory markers had increased to serum amyloid A (SAA) = 5032 mg/L (normal 0–30 mg/L) and white blood cell count = 24 × 10^9^/L (normal 5.45–12.65 × 10^9^/L). Despite aggressive treatment with anti-inflammatory medication (flunixin meglumine 1.1 mg/kg two times daily), signs of septicemia progressed and the horse was therefore euthanized 8 days after inoculation. Bacteremia with *S. aureus* was confirmed by culture on blood obtained 5 and 6 days after inoculation. Due to the course of the pilot study, inoculation dosages were reduced markedly and inoculation postponed to 4 days after wound creation.

### Clinical wound examinations and pH measurements

Wounds at each body location were examined on day 7, 10, 14, 17, 21, and 27 and scored according to amount of exudate (0/none, 1/mild, 2/moderate, and 3/severe), type of exudate (0/serous-hemorrhagic, 1/mucous, and 2/purulent), epithelialization (0/present and 1/not present/not visible), level of granulation tissue (0/below or in level with skin and 1/above skin level), quality of granulation tissue (0/even and 1/uneven), and color of granulation tissue (0/pink, 1/red, or dark red). Scores were totaled to create an overall wound score per body location per time point (range from 0 to 9).

Surface pH was measured on day 7, 14, 21, and 27 in wounds to be biopsied using a glass surface electrode (Blueline 27 pH, SI Analytics, Mainz, Germany)^28^ and a pH meter (PHM210 Standard pH Meter, Radiometer Analytical S.A., Villeurbanne Cedex, France), which were calibrated daily.

### Circumference and thermometry

Circumference of each wounded limb was measured between the two middle wounds using a sterile measuring tape on day 0, 4, 7, 10, 14, 17, 21, and 27. On the same days, skin temperature was recorded by thermometry after bandage removal using a handheld Raynger MX4 infrared thermometer (Raytek GmbH, Berlin, Germany) for 10 s at a 20 cm distance perpendicular to the skin. The temperature of the wounded skin on each wounded limb and thorax side was measured.

### Biopsy procedures

Biopsies (6–8 mm) from one randomly chosen wound at each location were collected at day 7, 14, 21, and 27. Before the biopsy procedure, wounds were gently cleansed with sterile gauze pads soaked in isotonic sterile saline. All wounds were only biopsied at one time point, and wounds were excluded from further analyses once they had been biopsied. The horses underwent sedation and nerve blocks as described above and had two biopsies collected: one biopsy (8 mm) from the wound margin (including granulation tissue and migrating epithelia/skin margin) and one biopsy from the center of the wound (6 mm). The margin biopsy was immediately fixed in 4% formaldehyde, transferred to 70% ethanol after 1 week, and finally embedded in paraffin. Biopsies from the wound center of inoculated wounds at day 7, 21, and 27 were used for bacteriological culture and were stored in sterile cryo tubes at 5°C for up to 40 h until further processing.

### Wound area measurements

At day 0, 4, 7, 10, 14, 17, 21, and 27 digital photography of all wounds was performed. Photographs were obtained using a Panasonic Lumix DMC-T220 camera with a 24-mm aspherical lens and 16 × optical zoom. In all photographs a flexible sterile ruler was included for calibration and for identification of location. The photographs were used for wound area measurements by using the image analysis software Fiji.^29^ Wounds that had been biopsied earlier were not included. Wound perimeter was traced three times and wound area recorded as the mean of these three tracings. The area of the granulation tissue, not including the advancing edge of epithelium if present, was measured.

### Bacterial culture

Wound biopsies were homogenized, sonicated, diluted in 10-fold series, and plated as 100 μL of the undiluted tissue suspension spread on a plate and 10 μL of the dilution-series 10^0^–10^4^ in spots on selective Pseudomonas Isolation Agar plates (Panum Institute Substrate Department, University of Copenhagen, Denmark) and LB plates added 6.5% NaCl (Panum Institute Substrate Department, University of Copenhagen, Denmark) selective for Staphylococci. The plates were grown aerobically for 18 h at 37°C. The CFU counts were calculated from the number of colonies in the countable dilutions.

### Bacterial aggregate detection by peptide nucleic acid fluorescence *in situ* hybridization and confocal laser scanning microscopy

To detect bacterial aggregates, peptide nucleic acid (PNA) fluorescence *in situ* hybridization (FISH) was performed as previously described after standard deparaffinization.^14,30^ Four replicates/sections of each margin biopsy were prepared with two different PNA probes; a specific *P. aeruginosa* Texas red/universal bacterial (BacUni) FITC (fluorescein isothiocyanate)-conjugated probe and a specific *S. aureus* Tamra/BacUni FITC-conjugated probe (both from AdvanDx, Inc., Woburn, MA). Furthermore, a nuclear counterstain was performed with 4′,6′-diamidino-2-phenylindole (DAPI).

Microscopic examinations were performed by confocal laser scanning microscopy (CLSM) (Axio Imager.Z2, LSM710 CLSM and LSM880 CLSM, Zeiss, Oberkochen, Germany) and the accompanying 3D reconstruction software (Zen 2010, version 6.0, Zeiss, Oberkochen, Germany) as described previously.^14^

Bacterial aggregates of >5 μm in diameter have been categorized as biofilms.^31^ To grade bacterial aggregates, a previously used^14^ categorical semiquantification was applied (grade 0, 1, 2 and 3). Tissue sections were systematically examined and bacterial aggregates were semiquantified according to the largest aggregate present targeted by either of the probes.

### Statistical analyses

All statistical analyses were performed in SAS Enterprise Guide 7.1 (SAS Institute, Inc., Cary, NC). Graphs were made in GraphPad Prism 5 (GraphPad Software, La Jolla, CA). When relevant, data are reported as mean ± SD or as parameter estimates with 95% confidence intervals.

Wound areas, pH values, culture results, wound scores, circumference, and thermometry measurements were analyzed using two-way ANOVA (factors being time and wound type [body position and inoculation status]) with repeated measurements taking account of correlations between all measurements on the same horse, closer correlation between wounds at the same position in the same horse, and furthermore an autoregressive error structure over time for each wound. For each outcome variable, limb wounds and thorax wounds were compared as well as inoculated versus noninoculated limb and thorax wounds, respectively. Blood parameters were analyzed using one-way ANOVA with autoregressive error structure over time. Standard model checks were applied for each analysis before processing. Even though wound scores were ordinal of nature, they were modeled as quantitative variables to obtain interpretable conclusions. Model checks showed satisfactory normality and variance homogeneity of residuals. Some parameters (wound areas, SA, and culture results) were log-transformed to achieve satisfactory model checks. For SAA and CFU/biopsy, it was necessary to give zero values a minimal value of 0.1 mg/L and 1 CFU, respectively, to be able to log-transform data.

Detection of bacterial aggregates (yes/no, corresponding to bacterial aggregate grade above 0/equal to 0) was analyzed using generalized estimating equations, to allow for correlations between findings on the same horse. For the thorax wounds, it was not possible to run a logistic regression model because of too few data points. Fisher's exact test was used to compare grading of bacterial aggregates per day for each body location, including thorax wounds. Level of significance was set at *p* < 0.05.

## Results

### Clinical observations, hematological, and biochemical evaluations

All surgical procedures were successful. One horse was slightly depressed and had slightly increased rectal temperature (38.7°C) on day 1. One horse became ill with strangles (airway infection with *Streptococcus equi ssp. equi*, confirmed on nasal swab culture) on day 4–10 and was isolated and treated with flunixin meglumine 1.1 mg/kg one to two times daily.

Four horses developed intermittent (1–3 days duration) mild lameness (1/5 or 2/5 AAEP grades) on one wounded limb. One horse developed 4/5 grade lameness on one hind limb on day 23 due to reaction to the injection of local analgesics; this was treated once with flunixin meglumine (1.1 mg/kg), and the lameness resolved the day after.

Pain scores remained low throughout the study; they ranged from 0 to 8 (maximum possible score is 30; a score of 9 or above indicates that additional pain medication should be provided) in the first 7 days after surgery and from 0 to 4 for the remainder of the study.

Concentrations of inflammatory markers in blood increased transiently after the surgical procedure, but did not change in response to the inoculation procedure (Supplementary Fig. S1). Blood results from the horse that developed strangles have been excluded in these analyses.

### Wound score, pH, circumference, and skin temperature

**Figure 1.**
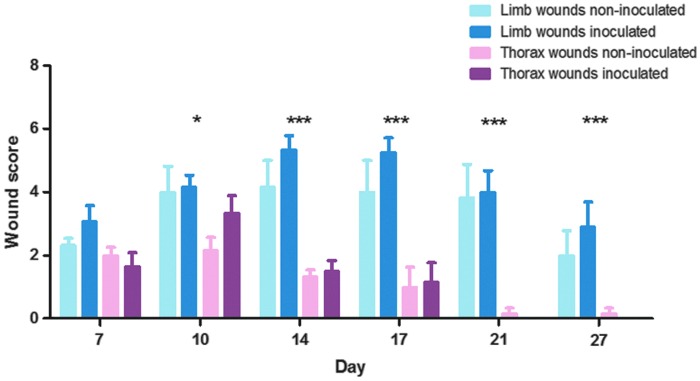
Wound score averages of experimental excisional limb and thorax wounds inoculated and not inoculated with *Staphylococcus aureus* and *Pseudomonas aeruginosa* on day 4. *Asterisks* indicate statistically significant differences between scores of limb and thorax wounds, ***p < 0.001 and *p < 0.05. There were no significant differences between inoculated and noninoculated wounds of the thorax or of the limbs, respectively. Six horses participated in the study, each with five wound areas (two limbs with inoculated wounds, one limb with noninoculated wounds, one thorax side with inoculated wounds and one thorax side with noninoculated wounds); each time point (day) thus includes six observations (one for each horse) per wound type except inoculated limb wounds, where each time point includes 12 observations (two for each horse; the exception being day 27, where only one inoculated limb wound was included per horse). Data are reported as mean ± SEM.

Granulation tissue appeared in all wounds on day 7. For limb wounds, the granulation tissue protruded above skin level from day 10 (in one horse), day 14 (in three horses), or day 17 (in two horses) onward. None of the thorax wounds developed exuberant granulation tissue at any time (Supplementary Fig. S2). Wound scores ranged for limb wounds between 0 and 8 (median = 4) and for thorax wounds between 0 and 5 (median = 1). Wound scores of limb versus thorax wounds developed significantly differently over time (*p* = 0.0002), with wound scores of thorax wounds being consistently lower than wound scores of limb wounds (Fig. 1). Wound scores were not significantly affected by inoculation (limb wounds: *p* = 0.067; thorax wounds: *p* = 0.48).

**Figure 2.**
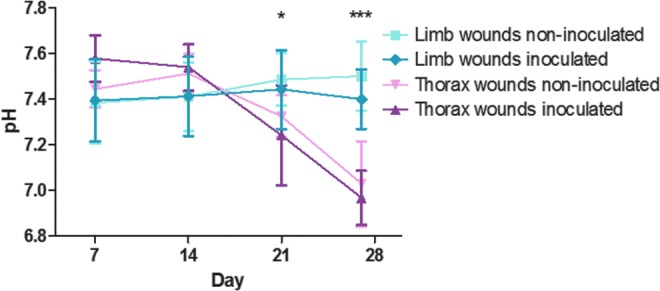
pH measurements of experimental excisional limb and thorax wounds inoculated and not inoculated with *S. aureus* and *P. aeruginosa* on day 4. *Asterisks* indicate statistically significant differences between pH of limb and thorax wounds, ***p < 0.001 and *p < 0.05. There were no significant differences in pH between inoculated and noninoculated wounds of the thorax or of the limbs, respectively. Six horses participated in the study, each with five wound areas (two limbs with inoculated wounds, one limb with noninoculated wounds, one thorax side with inoculated wounds, and one thorax side with noninoculated wounds); each time point (day) thus includes six observations (one for each horse) per wound type except inoculated limb wounds, where each time point includes 12 observations (two for each horse; the exception being day 27, where only one inoculated limb wound was included). Data are reported as mean ± SD

Surface pH measurements (Fig. 2) developed differently over time in thorax and limb wounds (*p* < 0.0001), with thorax wounds having significantly lower pH than limb wounds on day 21 (pH difference equal to 0.18, 0.004–0.35, *p* = 0.045) and day 27 (pH difference equal to 0.45, 0.26–0.64, *p* < 0.0001). Inoculation did not affect pH over time (*p* = 0.75).

Circumferences were similar in limbs with inoculated wounds and limbs with noninoculated wounds throughout the study period (*p* = 0.36). Temperatures of the wounded skin were not significantly higher for inoculated versus noninoculated wounds (*p* = 0.16).

### Area measurements

**Figure 3.**
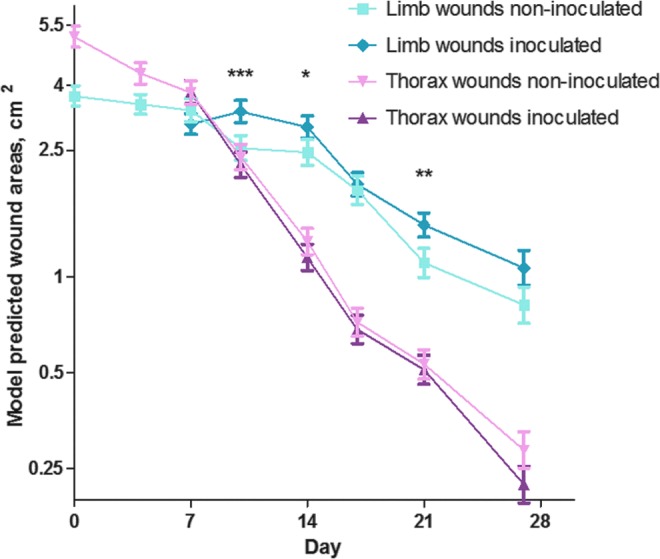
Wound areas (model predicted means and SEMs, log scale) of equine experimental excisional limb and thorax wounds inoculated and not inoculated with *S. aureus* and *P. aeruginosa* on day 4. *Asterisks* indicate statistically significant differences in the areas of inoculated and noninoculated limb wounds, ***p < 0.001, **p < 0.01, and *p < 0.05. For thorax wounds, no differences were detected between inoculated and noninoculated wounds throughout the study. Thorax wounds and limb wound were significantly different in size at all time points (not indicated in the figure). Six horses participated in the study, each with five wound areas of four wounds (two limbs with inoculated wounds, one limb with noninoculated wounds, one thorax side with inoculated wounds and one thorax side with noninoculated wounds). At each time point, every wound that had not previously been biopsied were included (day 4 + 7 = 20 wounds per horse, day 10 + 14 = 15 wounds per horse, day 17 + 21 = 10 wounds per horse, and day 27 = 4 wounds per horse).

Thorax wounds showed great immediate retraction and were 5.80 ± 1.22 cm^2^ shortly after creation; while limb wounds showed no retraction (area was 3.74 ± 0.52 cm^2^ shortly after wounds were created). Size of thorax wounds declined significantly faster than that of limb wounds (*p* < 0.0001, Fig. 3). In thorax wounds, inoculation did not affect size (*p* = 0.71), while in limb wounds size of inoculated and noninoculated wounds differed significantly over time (*p* < 0.0001), with inoculated wounds being larger than the noninoculated wounds from day 10 onward (Table 1).

**Table 1.** Percentage area difference of inoculated (with S. aureus and P. aeruginosa on day 4) relative to noninoculated limb wounds in an equine experimental excisional wound model

	*Day 7*	*Day 10*	*Day 14*	*Day 17*	*Day 21*	*Day 27*
Area difference of inoculated versus noninoculated limb wounds (95% confidence interval)	−10.0% (−22.0% to 0.8%)	31.2% (13.8–51.1%)	20.7% (2.6–42.0%)	5.2% (−12.8% to 27.1%)	32.1% (7.8–61.9%)	32.2% (−3.2% to 80.5%)
*p*-value	*p* = 0.070	*p* = 0.0002	*p* = 0.023	*p* = 0.59	*p* = 0.0075	*p* = 0.079

### Bacterial culture

**Figure 4.**
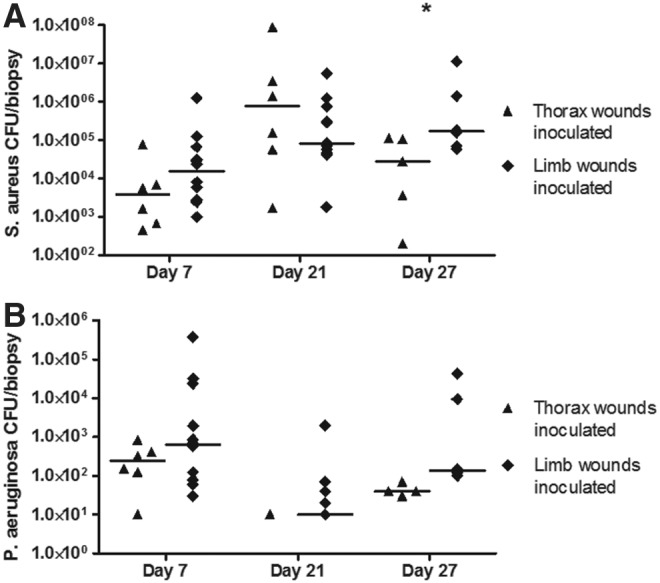
CFU counts of **(A)** *S. aureus* and (B) *P. aeruginosa* in biopsies from equine experimental excisional limb and thorax wounds inoculated with *S. aureus* and *P. aeruginosa* on day 4. *Asterisk* indicates statistically significant differences in CFU counts between limb and thorax wounds, *p < 0.05. Six horses participated in the study, each with two limbs and one thorax side with inoculated wounds; each time point (day) thus includes six observations (one for each horse) per thorax wound and 12 observations (two for each horse) per limb wound (the exception being day 27, where only one inoculated limb wound was included per horse). *Horizontal lines* show medians. CFU, colony-forming units.

CFU counts of *S. aureus* per biopsy (Fig. 4A) developed significantly (*p* = 0.047) differently in thorax and limb wounds over time. For both wound locations, *S. aureus* CFU increased from day 7 to day 21, but on day 27 CFU in thorax wounds decreased, while CFU in limb wounds increased. This resulted in 31 times (1.6–628, *p* = 0.026) higher CFU of the limb wounds on day 27 compared with thorax wounds. CFU counts of *P. aeruginosa* per biopsy (Fig. 4B) were generally low and were not detectable for all wounds on day 21 and 27; *P. aeruginosa* CFU counts did not differ significantly between thorax and limb wounds over time (*p* = 0.93).

### Bacterial aggregate detection

**Figure 5.**
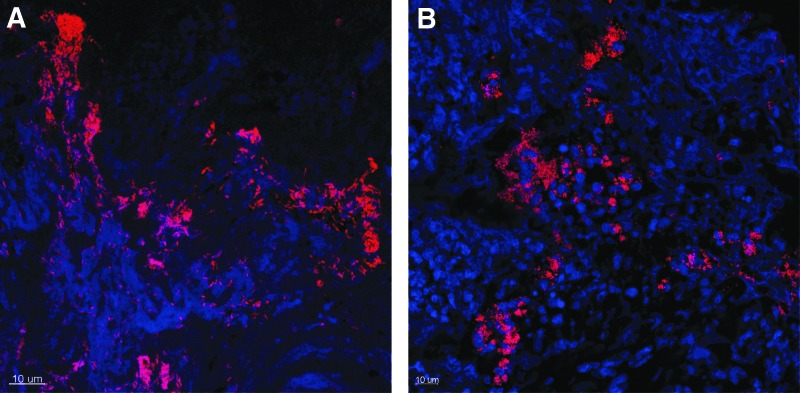
Confocal laser scanning microscopy images of bacterial aggregates in inoculated limb wounds in an equine experimental excisional wound model inoculated with *S. aureus* and *P. aeruginosa* on day 4. *Top* of image is toward surface of wound. Bacterial peptide nuclei acid probes are used to identify, respectively, *S. aureus* **(A)** and *P. aeruginosa* **(B)** as *red* bacterial cells. Equine immune cells appear *blue* due to DAPI nuclear counterstain. **(A)** Inoculated limb wound on day 7 (3 days after inoculation). Large aggregates (grade 3) of *P. aeruginosa* are seen below the wound surface. **(B)** Inoculated limb wound on day 27 (23 days after inoculation). Large aggregates (grade 3) of *S. aureus* are seen below the wound surface. Scale bar, 10 μm. DAPI, 4′,6′-diamidino-2-phenylindole

Bacterial aggregates were detected in most inoculated limb wounds on day 7 (91%), 14 (100%), and 21 (100%), and in fewer wounds (40%) on day 27 (Table 2 and Fig. 5). Bacterial aggregates were detected in some noninoculated limb wounds on day 7 (80%), 14 (50%), 21 (67%), and 27 (33%). Inoculated limb wounds had significantly higher odds of having bacterial aggregates present than the noninoculated limb wounds (odds ratio 7.1, 2.4–21.0, *p* = 0.0086). Furthermore, inoculated limb wounds had significantly higher bacterial aggregate grades on day 14 (*p* = 0.031) and day 21 (*p* = 0.0087) compared with noninoculated limb wounds (Table 2). In thorax wounds, bacterial aggregates were found in inoculated wounds only, and only on day 7 (83%), 14 (50%), and 21 (20%) (Table 3).

**Table 2.** Grading of bacterial aggregates and distribution of bacterial species detected in equine experimental excisional limb wounds inoculated or not inoculated with S. aureus and P. aeruginosa on day 4

*Grade/Inoculation Status*	*Day 7*		*Day 14*		*Day 21*		*Day 27*	
	*Inoculated*	*Noninoculated*	*Inoculated*	*Noninoculated*	*Inoculated*	*Noninoculated*	*Inoculated*	*Noninoculated*
0	1	1	0	3	0	2	3	4
1	4	2	2	1	3	3	1	0
2	3	1	5	2	7	0	1	2
3	3	1	5	0	0	0	0	0
Missing	1	1	0	0	2	1	1	0
Bacterial species	SA 70%;PA 50%; Other 20%	SA 74%; PA 0%; Other 50%	SA 83%; PA 25%; Other 8%	SA 67%; PA 0%; Other 33%	SA 90%; PA 10%; Other 20%	SA 100%; PA 0%; Other 0%	SA 100%; PA 0%; Other 0%	SA 50%; PA 0%; Other 50%
Fisher's exact test on grade per day	*p* = 1.0		*p* = 0.031		*p* = 0.0087		*p* = 1.0	

PA, *Pseudomonas aeruginosa;* SA, *Staphylococcus aureus.*

**Table 3.** Grading of bacterial aggregates and distribution of bacterial species detected in equine experimental excisional thorax wounds inoculated or not inoculated with S. aureus and P. aeruginosa on day 4

	*Day 7*		*Day 14*		*Day 21*		*Day 27*	
*Grade/Inoculation Status*	*Inoculated*	*Noninoculated*	*Inoculated*	*Noninoculated*	*Inoculated*	*Noninoculated*	*Inoculated*	*Noninoculated*
0	1	6	3	6	4	6	6	6
1	4	0	1	0	1	0	0	0
2	1	0	2	0	0	0	0	0
3	0	0	0	0	0	0	0	0
Missing	0	0	0	0	1	0	0	0
Bacterial species	SA 20%; PA 80%; Other 20%	SA 0%; PA 0%; Other 0%	SA 67%; PA 33%; Other 0%	SA 0%; PA 0%; Other 0%	SA 100%; PA 0%; Other 0%	SA 0%; PA 0%; Other 0%	SA 0%; PA 0%; Other 0%	SA 0%; PA 0%; Other 0%
Fisher's exact test on grade per day	*p* = 0.015		*p* = 0.18		*p* = 0.45		*p* = 1.0	

When comparing inoculated thorax and limb wounds, limb wounds had significantly higher odds of containing bacterial aggregates than thorax wounds (odds ratio 36.2, 3.8–348, *p* = 0.0018).

Aggregates consisted mainly of *S. aureus*, although a majority of the inoculated thorax wounds on day 7 contained *P. aeruginosa* (Tables 2 and 3). Multispecies aggregates were not detected, but within the same wound monospecies aggregates of two bacterial species were seen in several wounds. Most bacterial aggregates were detected below or at the wound surface close to the wound margin.

## Discussion

Establishment of an equine inoculated wound model with bacterial aggregates was successful, and inoculation affected healing of limb wounds negatively, but had no effect on thorax wounds. The differences in healing patterns of limb and thorax wounds observed in this study have been described in previous studies in horses (without inoculation), and it is generally accepted that limb wounds in horses are prone to impaired healing and chronic wounds.^8,14^ This difference in propensity for development of chronic wounds depending on body location is a further benefit of using the horse as model animal, as it allows researchers to study impact of bacterial inoculation on wounds with normal and wounds with poor healing within the same animal.

Wound healing of equine limb wounds is, similarly to wound healing in humans, mainly dependent on epithelialization with contraction playing a minor role.^7,8^ This makes equine limb wounds more similar to wounds in humans than rodent wounds, as healing in rodents occurs mainly by contraction.^4,32^ We created full thickness wounds, as these resemble the dermal losses seen in most human chronic wounds.^33^ Another advantage of our model is that neither mechanical contraptions of the skin nor medical or genetic modifications of the animals are needed to inhibit healing, as the healing in the limbs is naturally impaired. Such interventions are often necessary to induce impaired healing in commonly used rodent wound models.^4,32^ Yet, another advantage of using the horse as model animal is its size, which makes it possible to create several identical wounds on each body location. This means that the wounds can be considered the biological replicate, not the animals,^34^ thereby reducing the number of experimental animals in accordance with the Replacement, Reduction, and Refinement of animal experimentation. Furthermore, the many wounds mean that wounds can be followed over time and samples collected serially without euthanizing the animals. The welfare aspects of the equine excisional wound model (without inoculation) were recently assessed,^35^ and the model was found to cause mild discomfort and pain manifestations. Even with bacterial inoculation of several wounds in the present study, the horses showed only few and mild episodes of lameness and changes in general behavior and showed neither hematological nor biochemical responses in relation to inoculation. Therefore, we find the described model acceptable from an ethical and welfare point of view.

Several factors may explain why bacterial aggregates were more prevalent in inoculated limb wounds than in inoculated thorax wounds. One reason could be the less efficient local inflammatory response in limb wounds compared with body wounds of horses.^17,18^ In addition, body wounds have better blood supply and oxygenation,^16,36^ which are prerequisites for delivery of cells and factors to the wound bed needed for infection control and healing and for their function.^37^ This may explain why thorax wounds cleared the infection quite readily and healed with no complications. Yet, another explanation could be the decreasing pH of thorax wounds from day 21 and onward, as lower pH is known to be associated with enhanced immune response, better antimicrobial defense, and improved healing.^38^ Therapeutically lowering of pH has been shown to increase healing of human chronic wounds.^38^

Even in the noninoculated wounds, which were also maintained under sterile dressings, bacterial aggregates were still present in 80% of limb wounds on day 7, thereafter number of aggregates diminished, which is in line with previous findings in equine experimental excisional wounds.^14^ Apparently, bacterial aggregates naturally established in the noninoculated limb wounds were easier for the immune response to eradicate than aggregates formed in the inoculated wounds, suggesting that inoculation dosage, species, and virulence are important factors during establishment of wound infections.

Most aggregates were found just below the wound surface, which resemble the situation in most chronic wounds.^23^ This might also partly explain why resection of granulation tissue often is curative of wound infection and poor wound healing in equine limb wounds,^24^ and the same strategy is often used in chronic wounds in humans.^3^ The effect of resecting granulation tissue was not investigated in the present study, but this could be interesting in future studies to investigate causality between bacterial aggregates and the impaired healing pattern. Despite the markedly different healing patterns of thorax and limb wounds, the CFU counts of *S. aureus* were not statistically different between these two body locations on day 7 and 21; however, aggregates were found in all limb wounds but only in one thorax wound on day 21. This indicates that the immune response toward the bacteria and whether the bacteria form aggregates might be more relevant than the CFU counts. On day 27, the inoculated limb wounds had 31 times higher CFUs than the inoculated thorax wounds, indicating a better capability of the thorax wounds to clear the bioburden.

Effect of bacterial aggregates on healing occurred in limb wounds from day 10 onward, where inoculated wounds were larger than noninoculated ones. Bacterial aggregates affect healing negatively by eliciting ineffective and poorly coordinated inflammatory responses as well as causing collateral damage to the wound tissue.^39,40^ The delay between inoculation and affected wound healing could be related to the fact that bacterial aggregates need time to mature and develop, before they will negatively affect the healing.^41,42^

Even though inoculation affected healing in limb wounds negatively this was not reflected in the wound scores. This could be related to individual variation between horses or to the fact that clinical assessment of whether a wound is infected with bacterial aggregates or not is extremely difficult. A recent consensus guideline stated that aggregates should always be suspected in chronic wounds, even though specific clinical symptoms rarely testify to their presence.^3^ While we did not find increased periwound skin temperature near inoculated wounds, one study did report increased periwound temperatures in human chronic leg ulcers with confirmed infection.^43^

Detection of bacterial aggregates by use of PNA FISH and CLSM may be flawed, as aggregates are not evenly distributed in wounds^3,23,44^ and might be missed in the thin tissue sections. However, visual detection by either CLSM or scanning electron microscopy remains the gold standard of bacterial aggregate detection,^45,46^ and to increase the sensitivity, we investigated eight sections of 4 μm from each wound. Also, when performing bacteriological culture from wounds, there are risks of not detecting and underreporting bacterial numbers. We used center biopsies from the wounds for culture in this study, as biopsies are recommended for culture of bacteria from chronic wounds in humans, as swabs might fail to detect bacteria from deeper layers in the wounds.^47,48^ However, the heterogeneously distribution of bacteria within wounds is also a ubiquitous risk when sampling biopsies for culture.^31,49^

*P. aeruginosa* CFU counts were low in inoculated limb and thorax wounds; explanations for this could be that they were eradicated either by the host responses or the *S. aureus*, or that the inoculation procedure was unsuccessful. *P. aeruginosa* may also have escaped detection; a study of bacterial aggregate infected wounds in mice showed that the majority of bacterial aggregates were found in wound scabs,^50^ not in granulation tissue. Also, the cleansing procedure, however gentle it was, performed before sampling may have removed bacteria trapped in the often quite tenacious exudate overlying the wounds. In humans, *P. aeruginosa* is seldom found in acute and developing wounds, but is often preceded by other bacteria, such as Staphylococci, that might “prime” the wound bed before *P. aeruginosa* then settles as aggregates in chronic wounds.^2^ In this regard, our equine wound model resembles the clinical situation well, as *P. aeruginosa* did not settle in the wounds. A further development of the equine wound model could be to experiment with a staged inoculation procedure, postponing the *P. aeruginosa* inoculation until the limb wounds have a more chronic appearance after for example, 2–3 weeks.

Inoculation of wounds was done with planktonic-cultured bacteria to control bacterial numbers^4^; this is in contrast to other models, where preformed *in vitro* bacterial aggregates have been directly applied to wounds.^50^ It is unknown whether clinical wounds are infected with planktonic bacteria or aggregates and it might as well be a mixture of these types; however, the wound bed plays an essential role in bacterial aggregation and the change of the bacterial phenotype into the biofilm mode of growth.^3^

In conclusion, we succeeded in establishing an equine experimental excisional wound model with bacterial aggregates. Mainly *S. aureus* aggregates were detected, and the aggregates had a negative influence on the healing pattern of limb wounds, but not on that of thorax wounds. The model can be used to investigate many aspects of the influence of bacterial aggregates on wound healing, including mechanistic and pathophysiological events. Furthermore, the model can be used to test future novel treatments against bacterial aggregates.

## Innovation

Bacterial aggregates are a major cause of impaired wound healing and development of new relevant animal models is necessary to investigate effect of bacterial aggregates on wound healing. Our equine wound model with bacterial aggregate formation is in some aspects superior to other animal models, as both normal and impaired healing patterns toward inoculation and bacterial aggregates can be studied within the same animal over time. The established model of aggregate-infected wounds in horses gives us further possibilities to study the interactions between the immune response and the bacteria.

Key Findings

- In the established equine model, bacterial inoculation affected wound healing in limb wounds negatively, but had no effect on thorax wounds.
- Bacterial aggregates were significantly more often present in inoculated limb wounds than in noninoculated limb wounds and thorax wounds.
- Bacterial inoculation did not affect wound scores and could not be detected by clinical assessment of the wounds.
- This equine experimental excisional bacterial aggregate-infected wound model allows both normal and impaired wound healing to be studied over time.

## Supplementary Material

Supplemental data

Supplemental data

## About the Authors

**Stine Jacobsen, DVM, PhD**, is a diplomat of the European College of Veterinary Surgery, is Professor of Large Animal Surgery at UCPH, and an experienced researcher within equine experimental models, wound healing, and inflammation. **Elin Jørgensen, DVM**, is pursuing a PhD within equine chronic wounds and biofilm at UCPH. She has developed the equine experimental excisional bacterial aggregate-infected wound model based on her 10+ years' of experience with equine wound healing in clinical veterinary practice. **Lene Bay, MS, PhD,** is a postdoc in the human cutaneous microbiota at Costerton Biofilm Center, UCPH. **Lene Theil Skovgaard, MS,** is an associate professor at the Department of Biostatistics, UCPH, with many years of experience in analysis of repeated measurements and other correlated data. **Professor Thomas Bjarnsholt, DrMedSc, PhD** is head of a research group at Costerton Biofilm Center, UCPH, and is research manager of Department of Clinical Microbiology at Rigshospitalet and a pioneer researcher within biofilm and chronic infections.

## Abbreviations and Acronyms

AAEP: American Association of Equine Practitioners
BacUni: universal bacterial
CFU: colony-forming units
CLSM: confocal laser scanning microscopy
DAPI: 4′,6′-diamidino-2-phenylindole
FISH: fluorescence *in situ* hybridization
FITC: fluorescein isothiocyanate
LB: Luria–Bertani
OD_600nm_: optical density at 600 nm
PNA: peptide nucleic acid
SAA: serum amyloid A
UCPH: University of Copenhagen
