# Supplementary material for: An Equine Wound Model to Study Effects of Bacterial Aggregates on Wound Healing
Source: Adv Wound Care (New Rochelle). 2019 Aug 21;8(10):487–98. doi: 10.1089/wound.2018.0901 (PMC6709944; doi:10.1089/wound.2018.0901)
Supplement: Supplemental data [file Supp_Fig1.pdf]

Supplementary Data

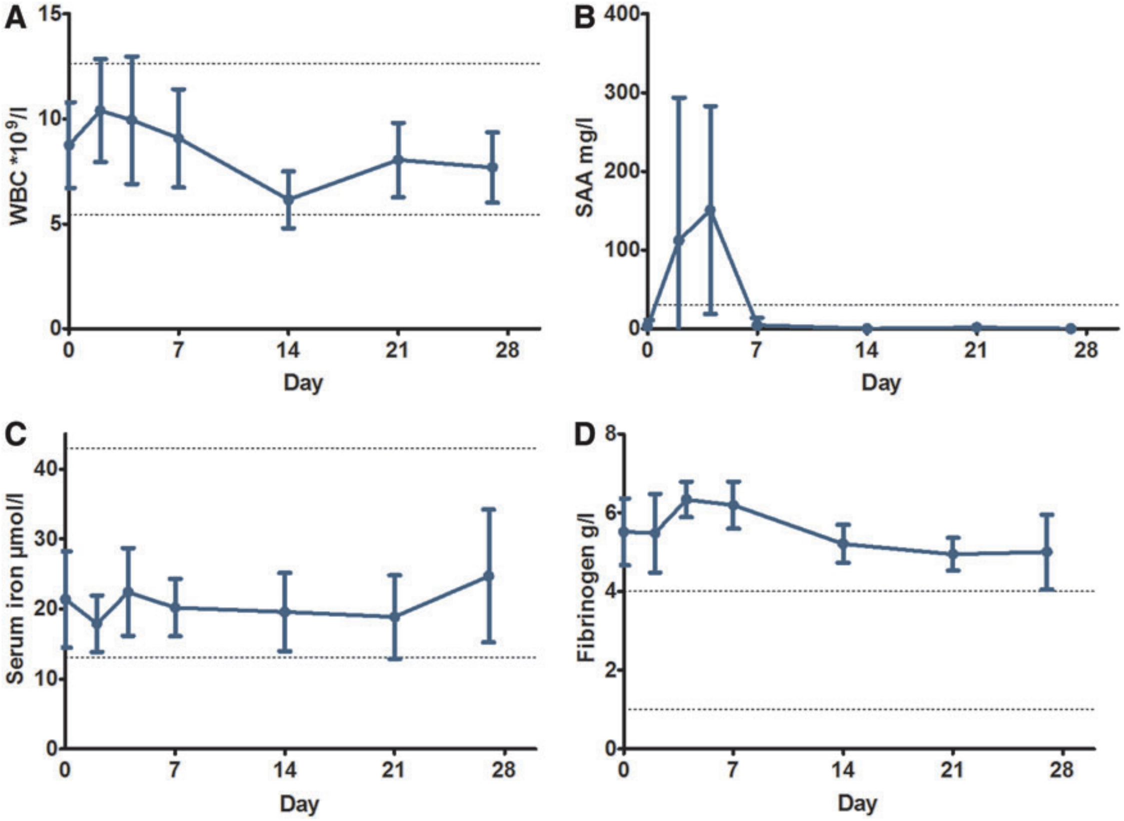

Supplementary Figure S1. WBC and biochemical markers for horses participating in an experimental excisional wound model with bacterial aggregate infection. **(A)** WBC stayed within reference levels (5.45–12.65 $\times 10^9$/L) throughout the study period, but increased significantly from 8.75 $\times 10^9$/L (6.23–11.27) to 10.40 $\times 10^9$/L (9.21–11.56, $p = 0.0083$) on day 0 to 2 and decreased significantly to 6.14 $\times 10^9$/L (4.29–7.99, $p = 0.0078$) on day 14. **(B)** SAA concentrations were within reference level (0–30 mg/L) on day 0 and increased significantly on day 2 (19 times, 1.2–307, $p = 0.037$) and 4 (133 times, 6.6–2680, $p = 0.0026$) to a peak (median) of 129 mg/L (range 0.1–349 mg/L) on day 4. **(C)** Serum ion concentrations stayed within reference values (13.1–43.0 μM) with no significant changes over time ($p = 0.42$). **(D)** Fibrinogen concentrations in the blood on day 0 (5.52 g/L; 4.63–6.40) were slightly above reference values (1.00–4.00 g/L) and increased significantly on day 4 to 6.34 g/L (5.67–7.02, $p = 0.019$). Data from five horses are included. Reference levels are indicated by *dotted lines*. SAA, serum amyloid A; WBC, white blood cell count.
