# Supplementary material for: An Equine Wound Model to Study Effects of Bacterial Aggregates on Wound Healing
Source: Adv Wound Care (New Rochelle). 2019 Aug 21;8(10):487–98. doi: 10.1089/wound.2018.0901 (PMC6709944; doi:10.1089/wound.2018.0901)
Supplement: Supplemental data [file Supp_Fig2.pdf]

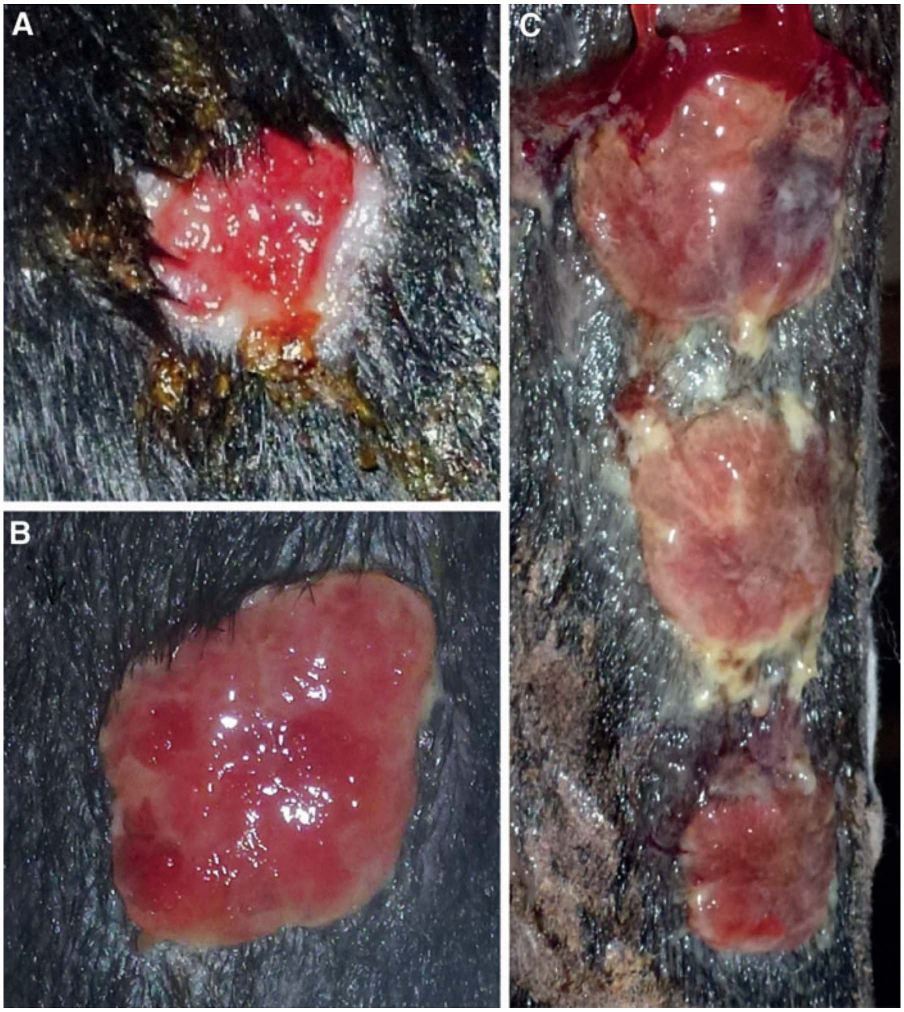

Supplementary Figure S2. Experimental excisional wounds healing by second intention in horses. Wounds were inoculated with *Staphylococcus aureus* and *Pseudomonas aeruginosa* on day 4. **(A)** Thorax wound on day 14 (10 days after inoculation) displaying normal healing with healthy granulation tissue, epithelial ingrowth, and sparse amounts of serous wound fluid. **(B)** Limb wound on day 14 (10 days after inoculation) displaying impaired healing with granulation tissue protruding above skin surface. The wound was gently cleansed before the photo was taken. **(C)** Limb wound on day 14 (10 days after inoculation) with large amounts of tenacious purulent exudate and granulation tissue protruding above the skin surface.
